# TAK-242 Ameliorates Hepatic Fibrosis by Regulating the Liver-Gut Axis

**DOI:** 10.1155/2022/4949148

**Published:** 2022-08-16

**Authors:** Sujie Liu, Juan Wu, Pingping Chen, Shadi A. D. Mohammed, Jingbo Zhang, Shumin Liu

**Affiliations:** ^1^Graduate School of Heilongjiang University of Chinese Medicine, Harbin, /150040 Heilongjiang, China; ^2^Institute of Traditional Chinese Medicine, Heilongjiang University of Chinese Medicine, Harbin, /150040 Heilongjiang, China

## Abstract

**Objective:**

The aims of this study were to investigate the impact of TAK-242 on the Toll-like receptor 4 (TLR4)/myeloid differentiation factor 88 (MyD88)/nuclear transcription factor-*κ*B (NF-*κ*B) signal transduction pathway in rats with hepatic fibrosis (HF) using the liver gut axis and to investigate the molecular mechanism of its intervention on HF.

**Methods:**

SPF grade SD male rats were randomly allocated to the control, model, and TAK-242 groups. For 8 weeks, the model and TAK-242 groups received 3 mL·kg^−1^ (the initial dose 5 mL·kg^−1^) intraperitoneal injections of 40% CCL_4_ olive oil solution. TAK-242 (5 mg·kg^−1^) was administered once a day for 5 days after modeling. The pathological alterations of liver and small intestine tissues in each group were observed using H&E and Masson staining. ELISA was used to measure serum levels of alanine aminotransferase (ALT), aspartate aminotransferase (AST), direct bilirubin (DBIL), total bilirubin (TBIL), interleukin-1*β* (IL-1*β*), interleukin-6 (IL-6), and tumor necrosis factor alpha (TNF-*α*). RT-qPCR was utilized to identify the mRNA expression level of IL-1*β*, IL-6, TNF-*α*, TLR4, MyD88, and NF-*κ*B in rat liver and small intestine tissues. The protein level of IL-1*β*, IL-6, TNF-*α*, TLR4, MyD88, and NF-*κ*B protein in rat liver and small intestine tissues was determined utilizing Western blot and IHC.

**Results:**

TAK-242 significantly reduced AST, ALT, TBIL, and DBIL expression in HF rats' serum (*P* < 0.01) and alleviated liver tissue injury. Hematoxylin-eosin (H&E) and Masson staining revealed inflammatory cell infiltration and fibrous proliferation in the liver and small intestine tissue in the model group and partial cell swelling in the TAK-242 group, which indicated a considerable improvement compared to the model group. RT-qPCR, Western blot, and IHC data indicated that TAK-242 reduced the IL-1*β*, IL-6, TNF-*α*, TLR4, MyD88, and NF-*κ*B expression in the liver and small intestine tissues of HF rats.

**Conclusion:**

TAK-242 might downregulate the TLR4/MyD88/NF-*κ*B signal pathway through the liver-gut axis, suppress the inflammatory response, and eventually alleviate HF in rats.

## 1. Introduction

Hepatic fibrosis (HF) is a wound healing process induced by chemical toxic damage, chronic hepatitis virus infection, autoimmune liver disease, alcoholism, and other variables that result in aberrant production and deposition of liver extracellular matrix. [1] Clinically, even once the cause is eliminated, HF persists and may progress to cirrhosis, hepatocellular cancer, and, eventually, liver failure leading to death [2]. In contrast to irreversible cirrhosis, a growing number of investigations have shown that HF is a dynamical and probably bilateral process with an intrinsic possibility for recovery and remodeling [3, 4], providing many new ideas for anti-HF mechanism research and clinical treatment. Multiple studies have revealed that the liver-gut axis is usually linked to the advancement of liver disease [5, 6]. The intestinal mucosal barrier function is weakened when intestinal homeostasis is disrupted, resulting in a large influx of intestinal endotoxins into the liver via the portal system [7]. These bacterial products aggravate the development of fibrotic lesions in liver tissue by stimulating natural immune receptors, such as Toll-like receptors (TLRs).

It activates myeloid differentiation factor 88 (MyD88) to release serine-threonine protein 1 kinase (IRAK 1 kinase) [8], which ultimately leads to the entry of nuclear transcription factor-*κ*B (NF-*κ*B) into the nucleus and ultimately activates downstream pathways involved in liver inflammation and fibrogenesis [5, 9]. It induces apoptosis in hepatic macrophages and the production of inflammatory molecules such as interleukin-1*β* (IL-1*β*), interleukin-6 (IL-6), and tumor necrosis factor alpha (TNF-*α*) [10–12], which exacerbate and destroy the intestinal barrier.

**Figure 1:**
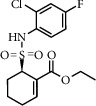
Chemical structure of TAK-242.

TAK-242 (Figure 1) is a small molecule of a toll-like receptor 4 (TLR4) that suppresses TLR4 activation by interacting directly with the intracellular domain of TIR [13]. Furthermore, TAK-242 shows hepatoprotective effects on Lipopolysaccharide/D-galactose (LPS/D-GalN)-induced fulminant hepatitis in mice [14], as well as suppression of TLR4 signaling to alleviate acute and chronic acute liver failure in animals. The TAK-242 has been demonstrated to minimize target organ damage and systemic inflammation in animal models [11], as well as ischemia/reperfusion injury in transplanted livers [15]; however, it is unclear if TAK-242 can specifically protect rats from HF through the liver-gut axis effects. This study evaluated the effect of TAK-242, a potential anti-inflammatory drug, in a CCl_4_-induced HF rat model, revealed its mechanism of action, and identified a potential therapeutic target for clinical HF therapy.

## 2. Materials and Methods

### 2.1. Animals and Experimental Protocol

SD male rats (SPF grade), weighing 230 ± 10 g (10 weeks old), were supplied by Heilongjiang University of Traditional Chinese Medicine's Experimental Animal Center (animal certificate number: SYXK (black) 2018-007). The rats were kept in the following conditions: room temperature of 22 ± 2°C, relative humidity of 40%-60%, good ventilation, alternating light and dark light for 12 hours, standard feed, and free drinking water, which were provided to the experimental animals, and the Heilongjiang University of Traditional Chinese Medicine Ethics Committee approved the experiment (approval number DXLL2020081601). The rats were randomly divided into three groups of eight rats each after one week of adaptive feeding: control, model, and TAK-242 (MedChemExpress, HYB0000050025). The model and TAK-242 groups received intraperitoneal injections of 40% CCL_4_ olive oil at 3 mL·kg^−1^ (5 mL·kg^−1^ for the first dose) [16, 17], while the control group received the same amount of olive oil twice a week for 8 weeks. After modeling, TAK-242 was administered once a day for 5 days with 10%DMSO + 90% (20% SBE-*β*-CD in saline) at a dosage of 5 mg·kg^−1^ [14, 18–20]. The control and model groups received an equal amount of normal saline by gavage.

### 2.2. Sample Preparation

The rats were anesthetized with a 3% pentobarbital sodium solution. Blood was taken from the abdominal aorta and centrifuged for 15 minutes at 3500 r/min. The serum was isolated and refrigerated at -80°C for analysis. The liver and small intestine tissues were separated, the left two lobes of the liver and a part of the small intestine tissue were preserved with 4% paraformaldehyde solution, and the remainder were placed in a cryopreservation tube for later use.

### 2.3. ELISA Detection Kits

Serum alanine aminotransferase (ALT), aspartate aminotransferase (AST), direct bilirubin (DBIL), total bilirubin (TBIL), interleukin-1*β* (IL-1*β*), interleukin-6 (IL-6), and tumor necrosis factor alpha (TNF-*α*) were measured using an ELISA kit according to Nanjing Jiancheng Institute of Biological Engineering kit instructions and then analyzed with a microplate reader (Thermo Company).

### 2.4. Histopathological Staining

#### 2.4.1. Hematoxylin-Eosin (H&E) Staining

Liver and small intestine tissues were fixed in 4% paraformaldehyde for more than 24 hours before being embedded using an alcohol gradient dehydration method. The embedded wax blocks were quickly sliced into sections 4 *μ*m thick. Finally, the slices were stained with hematoxylin and eosin (H&E), and the pathological changes were examined using an optical microscope (Nikon Eclipse E100).

#### 2.4.2. Masson Staining

For more than 24 hours, the liver tissue was immersed in 4% paraformaldehyde. The implanted wax blocks were quickly sliced into 4 *μ*m thick slices and dewaxed. The slices were then immersed in Masson solution and sealed with neutral gum. A microscope (Nikon Eclipse E100, Japan) was used to examine the tissue collagen fiber area.

### 2.5. Real-Time qPCR

Total RNA was extracted from liver and small intestine tissues utilizing the trizol technique and dissolved in enzyme-free water, according to the experimental protocol. The total RNA was then reverse transcribed into cDNA using a reverse transcription kit, the PCR reaction system was prepared using ROX Reference Dye II, and quantitative real-time PCR was performed using the MyiQ™ Optics Module monochrome real-time PCR detection system (BioRad, USA). Glyceraldehyde-3-phosphate dehydrogenase (GAPDH) was selected as the endogenous control. The relative quantification method was used to analyze the data, and the 2^−△△Ct^ method was used to analyze the data and determine the relative expression of mRNA.  Table 1 displays a list of sequencing primers.

### 2.6. Immunohistochemical (IHC) Staining

The immunohistochemistry detection was carried out in exact compliance with the immunohistochemical kit's instructions. Tissues from the liver and small intestine were embedded and sectioned, then dewaxed, hydrated, and cleaned. The sections were then blocked with 3 percent H2O2 and goat serum after being treated with a pH 6.0 sodium citrate buffer solution. Then, add TLR4 (ab22048, Abcam), MyD88 (ab133739, Abcam), NF-*κ*B (ab32536, Abcam), IL-6 (ab208113, Abcam), IL-1*β* (66737-1-Ig, Proteintech), TNF-*α* (ab1793, Abcam), ZO-1 (66452-1-Ig, Proteintech), and Claudin-1 (ab211737, Abcam) were stained for target proteins. DAB was observed and photographed under a microscope (motic, DMB5-2231P1 type) after dark color development. The brown color was positive. Image-Pro Plus 6.0 software was utilized for processing, and the integrated absorbance IA/area was used as the semiquantitative result of the detection index.

### 2.7. Western Blot Analysis

Liver or small intestine samples were ground and mixed with 1 mL of total protein extract to homogenize before centrifugation at 9000 rpm for 10 minutes to assess protein concentration. After boiling the protein for 3 minutes to denature it, the samples were put in a specified sequence for electrophoresis. When the bromophenol blue migrated to the bottom 0.5 cm of the separation gel, the gel glass plate was removed, and the polyvinylidene fluoride (PVDF) membrane was transferred. The electrophoresis was completed. After that, the electrotransfer membrane was blocked with 5% nonfat dry milk (PBS). At 4°C overnight, primary antibodies TLR4 (Ptgcn, 19811-1-AP), NF-*κ*B p65 (Ptgcn, 10745-1-AP), MyD88 (Ptgcn, 23230-1-AP), IL-1*β* (CST, #12242), IL-6 (CST, #12912), TNF-*α* (Ptgcn, 60291-1-Ig), and *β*-actin (Ptgcn, 66009-1-Ig) were mixed with 1 mL of enzyme-labeled secondary antibody. After washing the membrane with 2-3 mL of PBST, develop it with ECL reagent and assess the gray value of each band using Image-Pro Plus 6.0 software. The relative protein expression is determined by the ratio of the target protein band to *β*-actin.

### 2.8. Statistical Analysis

GraphPad Prism 8.0 was utilized for the analysis. The experimental data were presented as the mean ± standard deviation (*x* ± *s*), and they were tested for normality and variance homogeneity. When comparing two samples, the *t*-test was used, and when comparing multiple groups, one-way ANOVA was used. *P* < 0.05 indicating a statistically significant difference.

## 3. Results

### 3.1. TAK-242 Effect of Reducing HF in HF Rats

**Figure 2:**
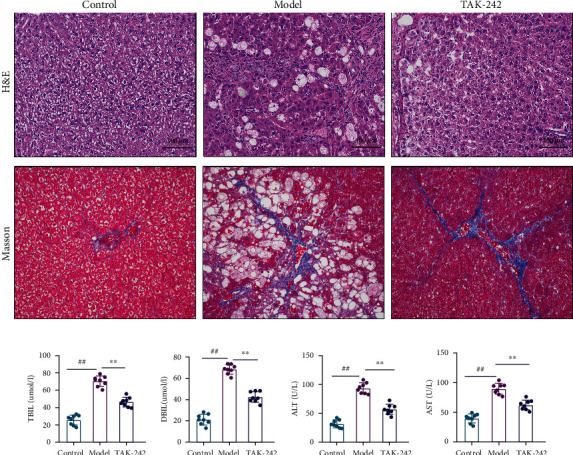
TAK-242 can alleviate HF-induced hepatic fibrosis in rat liver tissue. (a) Hematoxylin-eosin (H&E) staining and Masson staining of liver tissue (magnification, ×200). (b) Serum TBIL (*μ*mol/L). (c) Serum DBIL (*μ*mol/L). (d) Serum ALT (U/L). (e) Serum AST (U/L). *n* = 8. Compared with the control group, ^##^*P* < 0.01; compared with the model group, ^∗^*P* < 0.05 and^∗∗^*P* < 0.01. (b–e) Green, control; red, model; blue, TAK-242.

The hepatocytes of the rats in the control group were neatly arranged, with a clear structure, no degeneration or necrosis, no congestion in the hepatic sinus, and also no inflammatory cell infiltrate or fibrotic tissue proliferation, while in the model group, a considerable number of foam cells were found in the tissue of the rats and infiltration with a small number of lymphocytes and hyperplasia of connective tissue around a large number of venous vessels, accompanied by punctate necrosis of hepatocytes, nuclear fragmentation or lysis, enhanced eosinophilic cytoplasm, and rare bile duct hyperplasia. The TAK-242 group's liver tissue structure improved to varying degrees, connective tissue hyperplasia was greatly decreased, and the fibrous septum was significantly reduced, as shown in Figure 2. Furthermore, serum levels of AST, ALT, DBIL, and TBIL in the model group were significantly higher than in the control group (*P* < 0.01). The levels of AST, ALT, DBIL, and TBIL in serum of the TAK-242 group were significantly lower (*P* < 0.01) than those of the model group. The findings demonstrated that the CCL_4_-induced rat hepatic fibrosis model was effectively created.

### 3.2. TAK-242 Effect on Liver Inflammation in HF Rats

**Figure 3:**
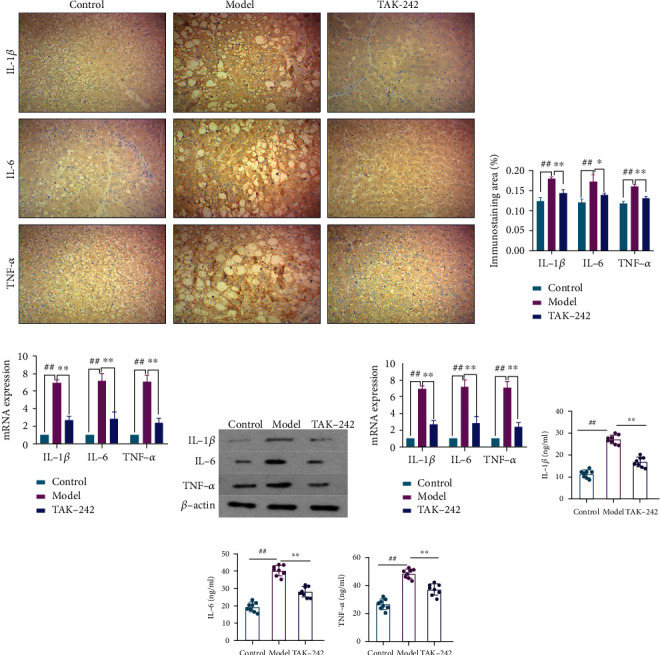
TAK-242 alleviated liver inflammation in HF rats. (a) IHC-stained liver sections (magnification ×200). (b) chromogenic intensity of proinflammatory cytokines. (c) RT-qPCR detection of hepatic proinflammatory cytokine expression level. (d, e) Western blot detection of hepatic proinflammatory cytokine protein expression. (f) Serum IL-1*β* (ng/mL). (g) Serum IL-6 (ng/mL). (h) Serum TNF-*α* (ng/mL). *n* = 8. Compared with the control group, ^##^*P* < 0.01; compared with model group, ^∗^*P* < 0.05 and^∗∗^*P* < 0.01. (f–h) Green, control; red, model; blue, TAK-242.

HF upregulates inflammatory factors such as IL-1*β*, IL-6, and TNF-*α* in the liver. Compared to the control group, the model group had significantly higher levels of IL-1*β*, IL-6, and TNF-*α* secretion and expression. In comparison to the model group, the TAK-242 group significantly reduced serum levels of IL-1*β*, IL-6, and TNF-*α*. Meanwhile, the protein and mRNA expressions of IL-1*β*, IL-6, and TNF-*α* were significantly lower in the TAK-242 group's liver tissue, Figures 3(a)–3(h). These findings suggested that TAK-242 might reduce the inflammatory response in HF rats.

### 3.3. TAK-242 Effect in Intestinal Barrier Function of HF Rats

**Figure 4:**
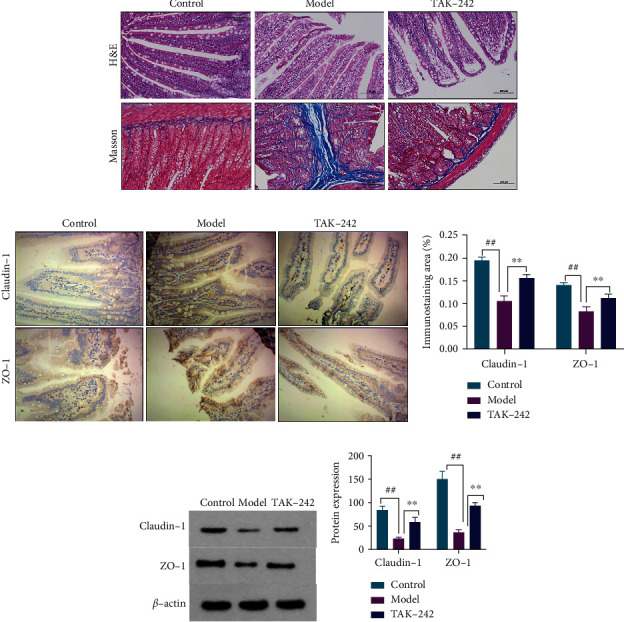
TAK-242 can regulate intestinal barrier function in HF rats. (a) Hematoxylin-eosin (H&E) staining of liver tissue, Masson staining (magnification, ×200). (b) IHC stained liver sections (magnification ×200). (c) Color intensity of intestinal wall permeability. (d, e) Western blot detection of intestinal wall tight junction protein expression. *n* = 8. Compared with the control group, ^##^*P* < 0.01; compared with the model group, ^∗^*P* < 0.05 and^∗∗^*P* < 0.01.

Claudin-1 and ZO-1 are two typical tight junction proteins that play important roles in the intestinal epithelium's tight junctions and permeability. The small intestine tissue cells in the control group's rats were well arranged, and there were no aberrant intestinal villi or cell infiltration. The mucosal layer of the small intestine tissue of the model group rats revealed a lot of epithelial edema, loose cytoplasm, and light staining, with dispersed lymphocyte infiltration, and a minor quantity of the epithelium was necrotic and shed, with condensed and stained nuclei. There were multiple mucosal layers and moderate edema of intestinal villi in the TAK-242 group's intestinal tissue, and the epithelium was separated from the lamina propria. The small intestine tissue of the TAK-242 group was improved to varying degrees as compared to the model group, and intestinal villus edema and lymphatic infiltration were dramatically reduced, Figure 4(a). In terms of mRNA and protein levels, claudin-1 and ZO-1 in the model group were significantly lower than those in the control group. In contrast, claudin-1 and ZO-1 in the TAK-242 group were significantly lower than in the model group (Figures 4(d) and 4(e)).

### 3.4. TAK-242 Can Reduce Intestinal Inflammation in HF Rats

**Figure 5:**
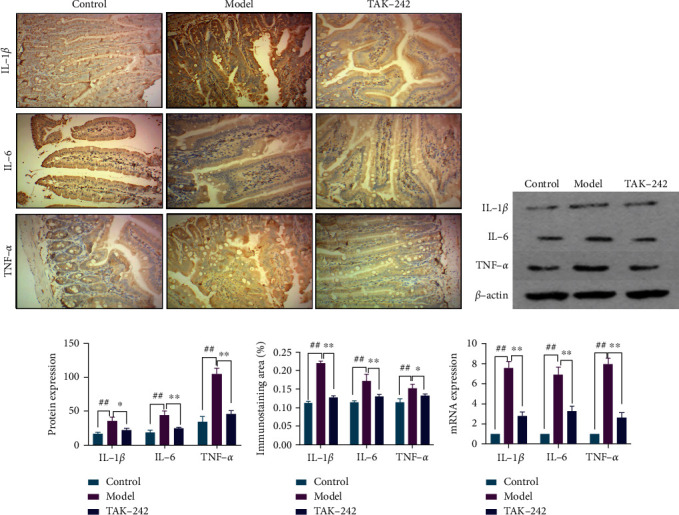
TAK-242 alleviates intestinal inflammation in HF rats. (a) Liver sections stained by IHC (magnification ×200). (b, c) Protein expression of intestinal proinflammatory cytokines detected by Western blot. (d) Color intensity of proinflammatory factors. (e) Expression of intestinal proinflammatory cytokines detected by RT-qPCR. *n* = 8. Compared with the control group, ^##^*P* < 0.01; compared with the model group, ^∗^*P* < 0.05 and^∗∗^*P* < 0.01.

When the intestinal epithelium's tight junctions and permeability are destroyed, the mucosal barrier function of the intestinal barrier is weakened, resulting in an inflammatory reaction in the intestinal tract that acts on the liver via the portal venous system and aggravates the pathological changes in the liver tissue. The expressions of IL-1*β*, IL-6, and TNF-*α* in the intestinal wall were measured to assess the influence of HF on the intestine. The mRNA and protein levels of IL-1*β*, IL-6, and TNF-*α* secretion and expression levels were increased significantly in the model group compared to the control group, while the secretion and expression levels of IL-1*β*, IL-6, and TNF-*α* in the TAK-242 group were significantly decreased compared with those in the model group (Figures 5(a)–5(e)**)**. These results indicate that TAK-242 may reduce intestinal inflammation caused by hepatic fibrosis.

### 3.5. Effects of TAK-242 on TLR4 Signaling Pathway Expression in HF Rats

**Figure 6:**
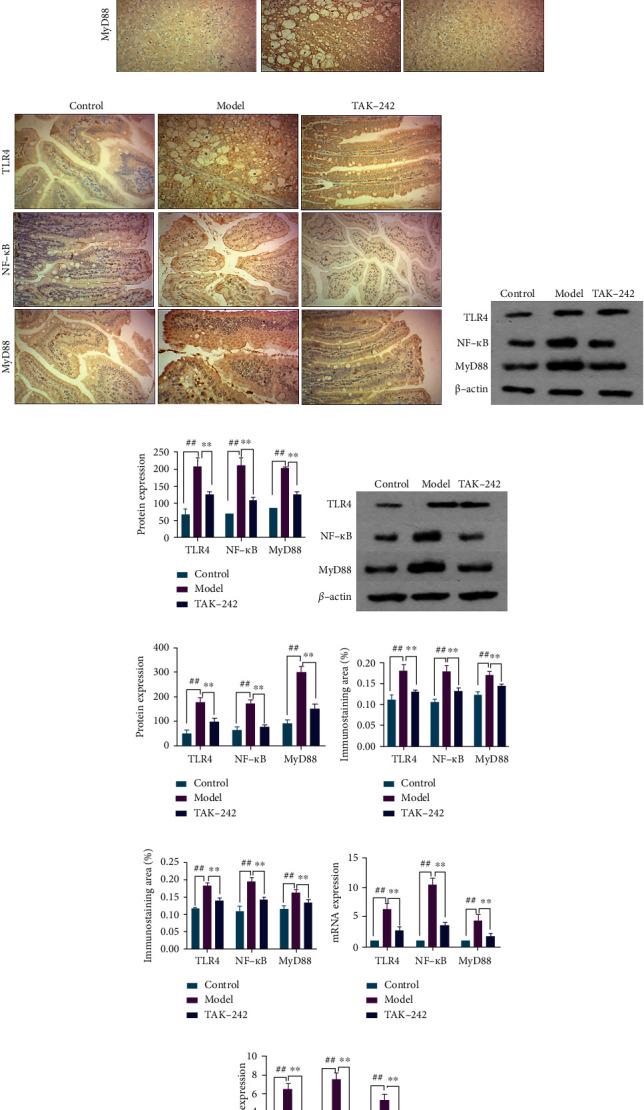
TAK-242's effect on TLR4 signaling pathway expression in HF rats. (a) IHC-stained liver section of liver tissue (magnification ×200). (b) IHC-stained intestinal section of intestinal tissue (magnification ×200). (c, d) Western blot detection of TLR4 signaling pathway protein expression in liver tissue. (e, f) Western blot detection of TLR4 signaling pathway-related protein expression in intestinal tissue. (g) Color intensity of TLR4 signaling pathway-related protein in liver tissue. (h) Color intensity of TLR4 signaling pathway-related protein in intestinal tissue. (i) RT-qPCR detection of TLR4 signaling pathway-related protein mRNA expression in liver tissue. (j) RT-qPCR detection of TLR4 signaling pathway-related protein mRNA expression in intestinal tissue. *n* = 8. Compared with the control group, ^##^*P* < 0.01; compared with the model group, ^∗^*P* < 0.05 and^∗∗^*P* < 0.01.

TLR4 is the initial barrier to bacterial detection in the gut and is a key component of gut innate immunity. It functions as an immunological recognition receptor on the cell surface as well as an intracellular transmembrane signaling protein. The MyD88-dependent signaling pathway dominates the signal transduction process following TLR4 activation. By simultaneously activating different intracellular signal adaptor molecules, NF-*κ*B downstream of the pathway is eventually activated to control the production of numerous inflammatory mediators. As a result, we identified key proteins associated with the TLR4 signaling pathway in the liver and small intestine tissue, respectively. The findings demonstrated that the model group had significantly greater TLR4, NF-*κ*B, and MyD88 secretion and expression levels than the control group. TLR4, NF-*κ*B, and MyD88 secretion and expression levels in the TAK-242 group were significantly lower than those in the model group, Figures 6(a)–6(j).

## 4. Discussion

The principal manifestation of liver fibrosis is an abnormal accumulation of extracellular matrix (ECM), which is typically regarded as an intermediate stage that may be cured or progress to cirrhosis and end-stage liver disease [28]. According to epidemiological data, more than one million individuals worldwide die from cirrhosis each year [29]. Cirrhosis is responsible for 9.2 fatalities per 100,000 people in the United States, according to epidemiological statistics from 2017 [30]. The burden of liver fibrosis raises not only the morbidity and mortality of end-stage liver disease but also the risk of extrahepatic disease. Modern research has established that the liver and the gut are not only physiologically connected not only in terms of structure (enterohepatic circulation) but also in terms of physiological functioning [31]. The findings of this investigation revealed that TAK-242 might inhibit the TLR4/MyD88/NF-*κ*B signaling pathway through the liver-gut axis, hence curing HF.

Researchers have found that [32–34] ALT and AST are essential enzymes in the liver, and their levels are directly associated to the progression of liver fibrosis and inflammation, and when liver cells are injured, enzymes enter the bloodstream via the cells, and the function of the liver cells to convert bilirubin is compromised. Inflammation in liver tissue destroys the capillary bile duct and impairs direct bilirubin excretion, resulting in elevated AST, ALT, TBIL, and DBIL levels. Our findings also revealed that serum AST, ALT, TBIL, and DBIL levels were greater in the model group than in the control group and that serum AST, ALT, TBIL, and DBIL levels could be significantly lowered following TAK-242 intervention. According to the pathological alterations in liver tissue, the model group had a high number of connective tissue and fibrous tissue hyperplasia and inflammatory cell infiltration, while the TAK-242 group's liver tissue improved to varied degrees. This result suggests that TAK-242 may alleviate the HF damage induced by CCL_4_.

Liu et al. [35] observed that bacterial translocation and elevated lipopolysaccharide levels in the gut stimulate TLR4 signaling and HSC activation in the liver. Meanwhile, claudins and occludins tight junctions play a vital role in the creation and maintenance of the intestinal epithelial barrier's integrity [36–38]. In our study, the intestinal mucosa tissues of HF rats in the model group were damaged, intestinal villi were diminished, and a considerable number of inflammatory cells were identified in the intestinal mucosa. Protein levels in the intestinal mucosa and villus were dramatically reduced after TAK-242 therapy. TAK-242 dramatically enhanced the protein levels of claudin-1 and ZO-1 in the small intestine of rats. TAK-242's antifibrosis activity in HF rats was suggested to be directly related to intestinal function.

In recent years, researchers have shown that inflammation is a major factor in the progression of liver fibrosis [39]. Liver injury may retain the active surface of HSCs and accelerate the migration of inflammatory cells to the injured liver, secreting a significant number of inflammatory mediators such as TNF-*α*, IL-6, and IL-1*β* to enhance the development of liver fibrosis [10–12, 40, 41]. TAK-242 was also observed to reduce the incidence of liver inflammation and fibrosis in Hu et al.'s research [42]. Similarly, the current research found that TAK-242 lowered the levels of inflammatory factors IL-1*β*, IL-6, and TNF-*α* in the liver. Furthermore, following TAK-242 treatment, the contents of inflammatory components in the small intestine of rats reduced, indicating that control of TLR4 expression may not only suppress the inflammatory response in the liver of HF rats. It also reduced the inflammatory response mediated by intestinal mucosal barrier damage in HF rats. These results revealed that TAK-242's anti-HF action in rats was connected to improved liver inflammation through the liver-gut axis.

The TLR4 receptor is a pattern recognition receptor. Its primary ligands are PAMP (LPS and Gram-negative endotoxin) and DAMP, which include cell death products (mitotic nucleosomes, histones, and HMGB1) [43, 44]. The research by Wu et al. [45] revealed that this membrane receptor was expressed on a wide range of nonsubstantial and substantial cells, including hepatocytes and hepatic stellate cells. Following ligand binding, the receptor dimers and recruits adaptor molecules, such as TIR-domain adaptor protein (TIRAP) MyD88 and TRIF-associated adaptor molecule (TRAM) TRIF, to create intracellular signaling complexes [45, 46]. MyD88-dependent signaling activates NF-*κ*B, while the TRIF-dependent pathway modulates interferon regulators, resulting in cytokine and interferon production [13]. Previous research by Naihua Hu et al. [47, 48] has demonstrated that TLR4/MyD88/NF-*κ*B may have an anti-inflammatory and hepatoprotective effect by decreasing ECM accumulation and inflammatory factor expression. It was consistent with our findings that TLR4, MyD88, and NF-*κ*B levels in HF rat liver tissue were elevated, whereas TLR4, MyD88, and NF-*κ*B levels were dramatically lowered following TAK-242 therapy. TLR4 is the initial barrier to bacterial detection in the gut and is a key aspect of gut innate immunity. It functions as a cell surface immunological recognition receptor and an intracellular transmembrane signaling protein. Furthermore, TLR4, MyD88, and NF-*κ*B levels in the small intestine of HF rats were significantly reduced after TAK-242 intervention, implying that TAK-242 improvement in HF rats may play a role by inhibiting the inflammatory response mediated by the TLR4/MyD88/NF-*κ*B signaling pathway via the liver-gut axis.

**Figure 7:**
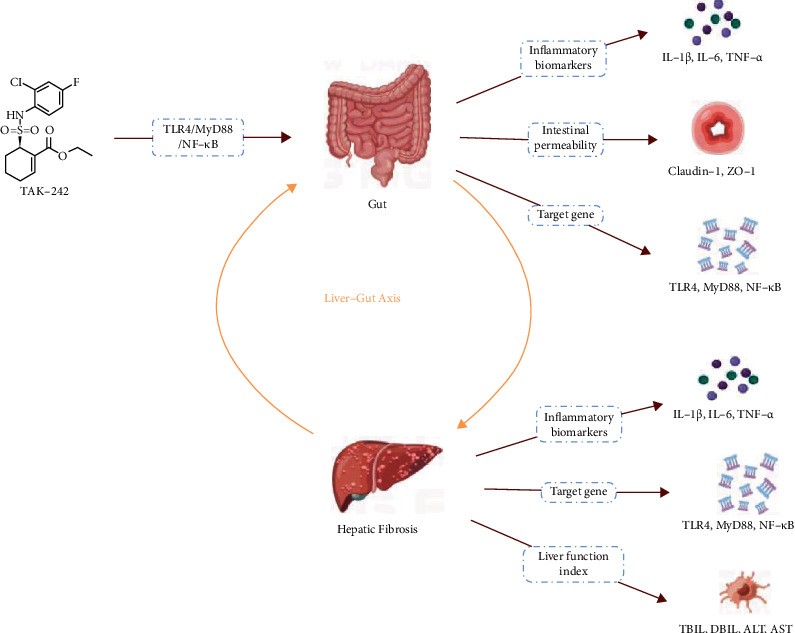
Flow chart of possible mechanisms of HF resistance in TAK-242, created using CmapTools.

Furthermore, changes in intestinal flora may help to explain the therapeutic mechanism; however, no research was conducted for this paper. As a result, the particular mechanism of TAK-242's therapeutic action on HF through the liver-gut axis requires additional investigation. Nonetheless, there is no doubting that this research has demonstrated the critical function of TAK-242 in the management of hepatic fibrosis. Nonetheless, this study has described an important role for TAK-242 in the treatment of liver fibrosis. The creation process using CmapTools is shown in Figure 7 [49].

## 5. Conclusion

In summary, this research illustrates that TAK-242 is implicated in CCL_4_-induced HF and inflammatory factor release in HF rats. TAK-242's anti-Hf effect is most likely achieved by liver-gut axis suppression of inflammation through the TLR4/MyD88/NF-*κ*B signaling pathway.

## Figures and Tables

**Table 1:** Real-time qPCR sequencing primers.

Gene	Forward	Reverse	Reference
GAPDH	TTTGAGGGTGCAGCGAACTT	ACAGCAACAGGGTGGTGGAC	[21]
NF-*κ*B	TGACGGGAGGGGAAGAAATC	TGAACAAACACGGAAGCTGG	[22]
TLR4	CCGCTCTGGCATCATCTTCA	CCCACTCGAGGTAGGTGTTTCTG	[23]
MyD88	TATACCAACCCTTGCACCAAGTC	TCAGGCTCCAAGTCAGCTCATC	[24]
TNF-*α*	CGTCGTAGCAAACCACCAAG	TTGAAGAGAACCTGGGAGTAGACA	[25]
IL-6	TAGTCCTTCCTACCCCAATTTCC	TTGGTCCTTAGCCACTCCTTC	[26]
IL-1*β*	TCGTGCTGTCGGACCCATAT	GGTTCTCCTTGTACAAAGCTCATG	[27]

## Data Availability

The data used to support the findings of this study are available from the corresponding author upon request.

## Abbreviation

ALT: Alanine aminotransferase
AST: Aspartate aminotransferase
DBIL: Direct bilirubin
TBIL: Total bilirubin
IL-1*β*: Interleukin-1*β*
IL-6: Interleukin-6
TNF-*α*: Tumor necrosis factor alpha
TLR4: Toll-like receptor 4
MyD88: Myeloid differentiation factor 88
NF-*κ*B: Nuclear factor kappa B
ZO-1: Membrane protein
Claudin-1: Transmembrane proteins.
